# Phase Method for Visualization of Hidden Dielectric Objects in the Millimeter Waveband

**DOI:** 10.3390/s19183919

**Published:** 2019-09-11

**Authors:** Igor V. Minin, Oleg V. Minin, Sergio Castiñeira-Ibáñez, Constanza Rubio, Pilar Candelas

**Affiliations:** 1 Nondestructive Department, Tomsk Politechnical University, 36 Lenin Avenue, Tomsk 634050, Russia; oleg.minin@ngs.ru; 2 Radiophysical Department, Tomsk State University, 30 Lenin Avenue, Tomsk 634050, Russia; 3 Centro de Tecnologías Físicas, Universitat Politècnica de València, 46022 Valencia, Spain; sercasib@upvnet.upv.es (S.C.-I.); crubiom@fis.upv.es (C.R.)

**Keywords:** imaging dielectric barrier, interferometry, millimeter wave

## Abstract

A method of detecting dielectric objects hidden behind an opaque barrier located on a reflective background, based on the distortion of interference fringes, is proposed in this article. Experiments conducted in the millimeter wavelength range demonstrated the effectiveness of the method under consideration, which does not require a holographic image reconstruction. Such a system can be classified as contour imaging.

## 1. Introduction

The ability to visualize metal objects behind a dielectric barrier has been shown by holographic methods [1] and direct radiovision [2,3,4,5]. In microwave, millimeter wave and THz, objects made from low loss dielectric material, including explosives, have a low reflectance, but they change the phase of the transmitted wave. We refer such objects as phase objects. Phase objects are also visualized for clearance [2]. However, it has not been possible to obtain dielectric object’s images against the reflecting body behind an obstacle yet.

Radio images of dielectric materials, including explosives, both in the method of direct radio imaging and in the recovery of holograms are obtained mainly due to the weak scattering of electromagnetic radiation on the sharp edges of objects [6]. At the same time, the intensity of the field scattered by well-reflecting metal objects significantly exceeds the intensity of the field scattered by dielectrics. As a result, dielectrics against the reflecting object practically disappear.

In this regard, there is a need for further research on the possibility of visualizing phase objects in the microwave range. In Reference [7], the principal possibility of visualization of phase objects in the “transmitted” mode in THz band is shown.

The task of visualization of phase objects is to transform the phase change of the object wave of electromagnetic radiation into intensity changes in the image of the object under study. The principal possibility of such visualization by holographic methods [7,8,9] with image restoration in the visible range from a reduced negative is shown. However, this procedure requires considerable mathematical processing and computational power [10,11].

The aim of this work was an experimental verification of the phase method of visualization of both dielectric and metal objects on a reflective background in the millimeter waveband. The dielectric object may be hidden under the dielectric target. This work proposed a new technique, which consisted of superposing a reference wave on the focused image of the object we were targeting, as it is described below. In this case, the so-called focused image hologram appeared in the recording plane [8,9], which was less sensitive to vibrations than other types of holograms. The reconstruction of an object could be achieved, for example, layer-by-layer for the same wavelength, so that three-dimensional images could be generated without scale distortions. However, with this method of reconstruction the usefulness of the phase method in real time becomes doubtful. It is interesting to be able to recognize phase objects directly from a hologram of a focused image [8,9] (which below we call interferogram for simplicity) in the process of its formation.

These interferograms of phase objects are formed on the background of a reflecting surface and consequently the characteristics of this surface, such as relief and roughness, will affect the resulting images. In the simplified case, if the object wave front and reference wave front are flat, and there is no dielectric object, then the field enhancement in the observation plane will be constant with a constant path difference (phase) between the object and reference waves and the distance to the reflecting surface. The introduction of the phase object into the object beam will lead to the appearance of interference fringes, which characterize areas of the same optical path difference. It could be noted that the proposed method of millimeter wave imaging differed from so-called phase-contrast imaging, for which a profile for free-space needs to be identified and used as a Reference [6].

The intensity distribution in the resulting interference pattern is described by the expression:

(1) I=A(1+cosΔϕ),

where Δϕ(x,y) is the phase difference between the object and reference waves. If the reflecting surface is non-planar or shifts during measurements, this will introduce an additional phase difference and distort the relief of the phase object. However, the measurement of the distance to the local areas of the reflecting surface at Δ*L* (see Figure 3) will lead to a phase change of 2*k*Δ*L* (where k = 2π/*λ* is the wave vector). It is obvious that a change in the thickness of the object Δ*L* leads to a change in the phase shift, equal to 2*k*Δ*Ln*, where n is the refractive index of the material of the phase object. Hence, the proposed method was *n* times more sensitive to changes in the thickness of phase objects than to changes in the relief of the reflecting surface.

In addition, it was assumed that, in the considered problem, the changes in the relief were rather smooth and the increase in the thickness of the phase object along its boundary had a sharper characteristic. All this should be considered when selecting the outlines of phase objects, even against the background of a relief object.

The motion of the entire object (a mannequin) as a whole could be compensated by the well-known method of extracting the reference wave from the object wave [8,9]. As a scanning system, we could use the rotation of the object itself for this purpose, placing it on a rotating table. In this case, the acquisition plane of interferograms and the reflective surface of the form, with respect to which the relief is implied, will be cylindrical surfaces.

## 2. Installation Description

**Figure 1:**
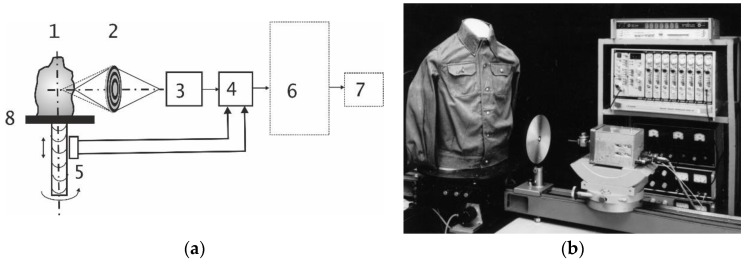
(**a**) Principal setup of the experimental plant: object (1), lens (2), phase-sensitive receiver (3), electronic block (4), rotation digital sensors (5), computer (6), monitor (7), rotating table (8); (**b**) the experimental interferometric setup for visualization of phase objects.

The experiments for ascertaining the validity of the proposed phase method were conducted following the block scheme shown in Figure 1a. An overview of the real test facility is shown in Figure 1b.

In the problem of detecting hidden objects under clothes a cylindrical aperture is usually used [12,13]. Scanning over a cylindrical aperture has some advantages over plane apertures, because of this particular task, as the distance to the sensor stays minimal during a body scan and each side of the body is illuminated. Processing holograms registered over a cylindrical aperture application of fast Fourier transform algorithms is not as straightforward as it is for plane holograms. A common approach usually involves signal interpolation over an equidistant rectangular spatial grid or application of non-uniform Fourier transform [10,12].

The object (a mannequin, numbered as 1 in the experimental block scheme) was placed on a rotating table (8 in Figure 1a), on which the object was scanned [10]. The object was irradiated by a focused beam of electromagnetic waves of the millimeter range with the wavelength of 2 mm, using a diffractive lens (2 in the experimental block scheme, Figure 1a), based on the phase zone plate [10,14]. The radiation reflected from the object was focused by the same lens (2 in the experimental block scheme, Figure 1a) on the same receiving and transmitting antenna of the phase-sensitive receiver (scatterometer [15,16] 3 in the experimental block scheme, Figure 1a, which emitted the millimeter wave signal and receive the return signal using a common lens 2). The diameter of the zone plate was 200 mm, the distance to the object was 220 mm, and the distance to the receiver was 120 mm. The resolution achieved in the experiment was approximately 2 mm (which corresponds to around two wavelengths). The dependence of the focal length of the zone plate on the radiation frequency allows, normally, frequency scanning in depth [3,5], which was specifically verified in this experiment. During the scanning, such a scheme was immediately noticed, an exact conjugation of the focus areas of the illuminator and the receiver. The signal allocated at the receiver’s output, carrying information about the phase of the wave reflected from the current point of the object, was fed to an analog-to-digital converter that was rigidly synchronized with the rotating table with horizontal and clock sync pulses. The digitized signal was the output to the video monitor (7 in the experimental block scheme, Figure 1a).

**Figure 2:**
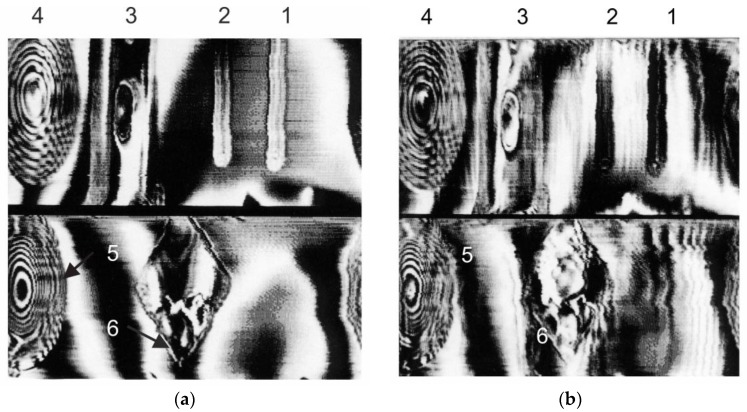
The images of the targets on the object under the text: metal rods (1,2), a polyethylene bar with an oval hole in the center (3), a dielectric lens (4), dielectric zone plate (5) and a square of polystyrene (6): without (**a**) and with plastic sheet (**b**) - see description above. The images are scaled on the horizontal axis.

A mannequin, 1 in Figure 1a was either a plastic form covered with conductive paint to provide reflection of electromagnetic waves, or a straight metal cylinder. The following objects placed on a reflective background, to detect them, were used (Figure 2a): metal rods of 20 mm (1, 2), a bar of polyethylene (50 mm thick) with an oval hole in the center (3), a lens of the same material with a smoothly varying thickness from 5 to 35 mm (4), a phase zone plate (5) with radio-optical properties similar to the aforementioned lens and a square of polystyrene (refraction index, *n* ≈ 1.5) with a thickness of 25 mm (6). A cotton shirt, a suit jacket with lining and a lampshade from a desk lamp made of 0.5 mm thick plastic and covered with synthetic fabric were used as dielectric barriers to hide an object (Figure 2b).

The rotating table made a full turn, remaining at the same level, and then (within the range of about 45 degrees) made a rise of 0.5 mm.

The phase sensitive transceiver was made according to the homodyne scheme with sinusoidal frequency modulation and with a combined receiving-transmitting path. The path of the emitted signal was mixed (without phase adjustment) on a quadratic detector with the received signal reflected from the object, thereby forming an artificial cylindrical reference wave. The threshold sensitivity of the receiver was 10–12 watts; the dynamic range of the receiver was about 30 dB.

## 3. Results and Discussion

All images in Figure 2 have 256 × 128 pixels. The horizontal spacing between readings was twice the vertical pitch for greater coverage of the object angle. For this reason, all objects appeared compressed horizontally. The angle of the object in each photo was equal to π degree.

Figure 2a shows a scan of a cylindrical interferogram of various objects against a metal cylinder: metal rods (1,2), a bar of polyethylene with a hole in the center (3), a zone plate (4), lenses (5) and a flat square (6) of polystyrene. In Figure 2a items were open, in Figure 2b were closed with a two-layer barrier made of plastic (0.5 mm thick) and synthetic fabric.

At the same time, it was noticeable that with an increase in the thickness of the barrier, sharp contours diffused and smashed (Figure 2b) which may have been due to the defocusing effect of a thick refractive medium on a beam of electromagnetic waves converging at a large angle. The defocusing effect increased with an increase in the thickness of the dielectric barrier, its refractive index and the angle of convergence of the beam (the aperture angle of the focusing system).

**Figure 3:**
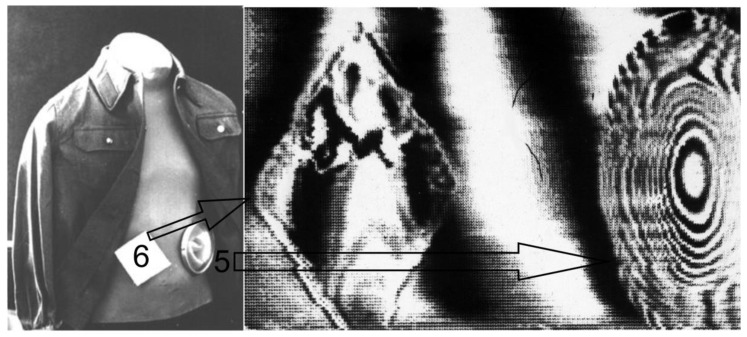
A mannequin with two dielectric objects: a dielectric lens (5) and a square of polystyrene (6) and correspondent images (zoom from Figure 2).

For a more visual presentation of the experimental results and understanding, Figure 3 shows the location of two dielectric objects on a mannequin in the visible wavelength range and their image obtained using the method under consideration.

From Figure 2 and Figure 3 it was obvious that the phase image of the dielectric lens and the zone plate looked the same because of the multiplicity of the phase incursion of 2π degree.

**Figure 4:**
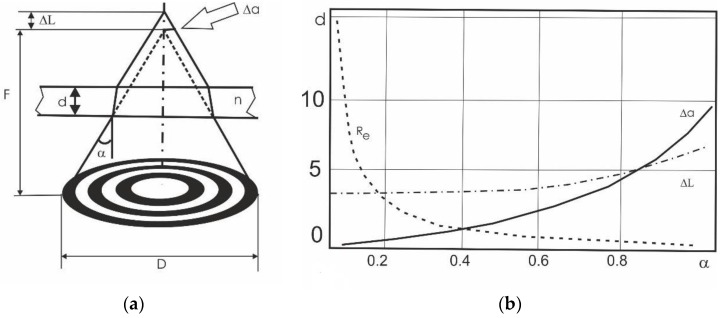
Effects of defocusing in the imaging of a hidden object: geometrical optics scheme (**a**) and defocusing parameters vs. aperture angle (**b**).

From Figure 4 it was easy to obtain expressions for the displacement of the focused point in depth (*Lc*), defocusing in depth (Δ*L*) and defocusing in the transverse direction (Δ*a*) for the case of a beam focusing through a plane-parallel transparent medium:

(2) $$\Delta a=d\mathrm{tg}\alpha \left(1-\frac{\cos\alpha }{\sqrt{n^{2}-\sin^{2}\alpha }}\right),$$

(3) $$\Delta L=\frac{\Delta a}{\mathrm{tg}\alpha }=d\left(1-\frac{\cos\alpha }{\sqrt{n^{2}-\sin^{2}\alpha }}\right),$$

(4) $$L_{c}=\Delta L_{\alpha =0}=d\frac{n-1}{n},$$

where *d* is the thickness of the plane-parallel dielectric, *n* its refraction index, α=arctg(D/2L) the aperture angle of the optical system of diameter *D* with its distance from the object L.

Figure 4b shows functions plots of Δa(α), ΔL(α) according to Equations (2)–(4) and Airy spot radius *R*e, where we set *d* = 10 mm, *n* = 1.5, λ = 2 mm. The results from Figure 4 showed that imaging through a transparent barrier, increasing the aperture of the optical system led to an improvement in resolution only to a certain limit. As the aperture increased further, the resolution deteriorated. The optimal aperture angle for the calculated case was *α* ≈ 180. This effect can also be partially compensated by refocusing the optical system by *Lc*.

It can be seen from Figure 2 and Figure 3 that the phase method made it possible to detect radio transparent objects both on a smooth reflective background and on a relief pattern behind a dielectric barrier made of plastic and fabric.

It should be mentioned that the proposed method of visualization of phase objects allows, in the presence of a “fast” scanning system (for example, a matrix of microwave receivers), to compensate the movement of an object as a whole by using a subject wave as a reference wave. This indicates the prospects of the proposed method and the need for its further development.

## 4. Conclusions

A phase method called “interference imaging” was proposed and tested for visualization of hidden dielectric objects on a reflective background in the millimeter waveband. It allows the direct imaging of a contour of a hidden dielectric target. An experimental setup for the investigation of phase method has been created and tested. A series of experiments with dielectrics made of polyethylene, polystyrene and various fabrics have been carried out. The possibility of visualization of dielectrics on the reflecting background by the phase method, as well as the prospects of the proposed method, were shown. The phase interferometric method made it possible to detect radio-transparent objects both against a smooth reflecting background and against a profiled background hidden behind a dielectric shield of plastic and fabric. In further works, we intend to combine images obtained in the millimeter range with the image obtained using a video camera. This will allow the more accurate determination of the location of hidden dielectric objects. We also plan to use a matrix or receivers to increase the speed when obtaining images in the millimeter range.
